# tRNA‐derived fragment tRF‐1020 ameliorates diabetes‐induced retinal microvascular complications

**DOI:** 10.1111/jcmm.17555

**Published:** 2022-09-20

**Authors:** Cong Ma, Jianling Du, Xiang Ma

**Affiliations:** ^1^ Department of Ophthalmology First Affiliated Hospital of Dalian Medical University Dalian Liaoning Province China; ^2^ Department of the Endocrinology First Affiliated Hospital of Dalian Medical University Dalian Liaoning Province China

**Keywords:** angiogenic function, retinal microvascular complication, tRNA‐derived RNA fragment, Wnt signalling

## Abstract

Transfer RNA (tRNA)‐derived fragments are the non‐coding single‐stranded RNAs involved in several physiological and pathological processes. Herein, we investigated the role of tRF‐1020, a tRNA fragment, in diabetes‐induced retinal microvascular complications. The results showed that the levels of tRF‐1020 expression were down‐regulated in diabetic retinal vessels and retinal endothelial cells following high glucose or H_2_O_2_ stress. Overexpressing tRF‐1020 led to decreased endothelial cell viability, proliferation, migration, and tube formation and alleviated retinal vascular dysfunction as shown by decreased retinal acellular capillaries, vascular leakage, and inflammation. By contrast, tRF‐1020 silencing displayed the opposite effects. tRF‐1020 regulated endothelial angiogenic functions and retinal vascular dysfunction by targeting Wnt signalling. Moreover, the levels of tRF‐1020 expression were reduced in aqueous humour and vitreous samples of the patients with diabetic retinopathy. Collectively, tRF‐1020 is a potential target for the diagnosis and treatment of diabetic retinopathy.

## 1 INTRODUCTION

Diabetic retinopathy (DR) is a major cause of visual loss among the working‐age people. It is also one of the most common microvascular complications of diabetes.^1^ The pathogenesis of DR is tightly associated with increased vascular permeability, vascular occlusion, and neovascularization.^2^ Diabetes‐induced retinal microvascular complications can lead to vitreous haemorrhage, tractional retinal detachment, neovascular glaucoma, and eventually blindness.^3^ Current treatment methods include intravitreal pharmacologic agents, panretinal laser photocoagulation, and vitreous surgery, which can retard vision loss to a certain extent. However, these methods can also cause several side effects, such as rare infectious, retinal detachment, and increased intraocular pressure.^4^, ^5^ Further studies are still required to search for the potential targets or new alternative therapies to prevent or delay the progression of DR.

Transfer RNAs (tRNAs) are a class of noncoding RNA transcripts corresponding to the delivery of specific amino acids to ribosome for translation.^6^ tRNAs can be cleaved by specific nucleases (e.g., Dicer and angiogenin) to produce tRNA‐derived small RNAs (tsRNAs), including tRNA‐derived fragments (tRFs) and tRNA‐derived stress‐induced RNAs (tiRNAs).^7^ tRFs are derived from the mature or primary tRNAs, which can be classified tRFs into five types by their derivations: tRF‐1, tRF‐2, tRF‐3, tRF‐5, and i‐tRF. tiRNAs are cleaved from the anti‐codon loop of tRNAs under stress such as oxidative stress, hypoxia, and heat shock. Previous studies have revealed that tRNA fragments participate in gene regulation in an RNAi manner or RNA silencing manner.^8^ tRFs and tiRNAs have been reported to be enriched in the body fluids. The composition and quantity of these RNAs are tightly dependent on cell type and disease condition. Stress‐induced tRFs and tiRNAs have been discovered to play a key role in cancers, metabolic diseases, and nervous disorders.^9^, ^10^ Nevertheless, the expression and clinical significance of tRFs and tiRNAs in DR remains unclear.

In this study, we mainly investigated the role of tRF‐1020, a tRNA fragment, in diabetes‐induced retinal vascular complications. The result showed that the levels of tRF‐1020 expression were significantly down‐regulated in diabetic retinal vessels, vitreous samples, and aqueous humour samples of DR patients. Increased tRF‐1020 expression could retard the progression of retinal vascular dysfunction via the inactivation of Wnt signalling. tRF‐1020 may serve as a potential target for the diagnosis and treatment of DR.

## 2 MATERIALS AND METHODS

### 2.1 Ethics statement

Animal experiments were conducted according to the guidelines of the Statement for the Use of Animals in Ophthalmic and Vision Research (ARVO) and approved by the Animal Care and Use Committee of the author's institute (No. 20190302–42). The clinical samples were collected according to the Declaration of Helsinki. All included patients were given the informed consent.

### 2.2 Streptozotocin‐induced diabetic mice

Eight‐week‐old male C57BL/6 mice were housed under a controlled temperature with free access to water and food. The diabetes was induced with intraperitoneal (i.p.) injection of 50 mg/kg streptozotocin (STZ, 572201, Sigma‐Aldrich) or vehicle (0.1 M citrate buffer, pH 4.5) for 5 consecutive days. The levels of fasting blood glucose (FBG) were detected by an Accu‐Chek Performa glucometer system (Roche Diagnostic). The mice with FBG levels greater than 16.7 mmol/ L were considered to be hyperglycaemic.^11^

### 2.3 Cell culture and transfection

Human retinal vascular endothelial cells (HRVECs) were cultured in Dulbecco's modified Eagle's medium (DMEM, Gibco) containing 10% fetal bovine serum, 1% penicillin–streptomycin solution (PS, Gibco), and endothelial cell growth supplement (Sigma Aldrich Corp) at 37 °C with 5% CO_2_ in a humidified atmosphere. One day prior to transfection, they were seeded into the 24‐well plates. When the confluency reached at 85%, HRVECs were transfected with negative control (NC) mimic, tRF‐1020 mimic, tRF‐1020 inhibitor, negative control (NC) inhibitor, or left untreated (Ctrl) using lipofectamine 6000 (Beyontime) following the manufacturer's protocol. After 12 h, the medium was replaced and cells were cultured at 37°C for additional 36 h for the subsequent studies. The sequences were shown below: tRF‐1020 mimic, 5’‐UCGGAGGCUUUGUUUU‐3′; NC mimic, 5’‐UCGCUUGGUGCACGUCGGG‐3′; tRF‐1020 inhibitor, 5’‐AAAACAAAGCCUCCGA‐3′; NC inhibitor, 5’‐UCUCCGAACGUGUCACGUU‐3′. Inhibitors and miRNA mimics were synthesized by GenePharma. During transfection, the concentrations of mimics and inhibitors were 30 nM.

### 2.4 RNA extraction and quantitative reverse transcription polymerase chain reaction (qRT‐PCR)

Total RNAs were extracted using the TRIzol reagent (Ambion). Quantitative reverse transcription polymerase chain reactions (qRT‐PCRs) were conducted on an Applied Biosystems StepOne Real‐time system (Applied Biosystems). These RNAs were pre‐treated by rtStar™ tRF&tiRNA Pretreatment Kit (Arraystar) and then reversely transcribed into cDNAs using the rtStar™ First‐Strand cDNA Synthesis Kit (Arraystar). The reactions were carried out using the SYBR Premix Ex Taq II kit (Takara). The 2^−ΔΔCt^ method was used for quantifying tRF‐1020 expression normalized with U6.

### 2.5 Cell viability assay

Cell viability was determined by Cell Counting Kit‐8 (CCK8) assay (Dojindo, Kumamoto, Japan) according to the manufacturer's protocols. Briefly, HRVECs were seeded into 96‐well plates at a density of 3 × 10^3^ cells/well with 100 μl of 10% FBS DMEM medium. After the required treatment, 10 μl of CCK‐8 solution was added to each well of the plate and then incubated for 3 h. The absorbance at 450 nm wavelength was detected by a microplate reader (Molecular Devices).

### 2.6 Cell proliferation assay

Cell proliferation was determined by ki67 immunofluorescence staining. Briefly, HRVECs were seeded in a 24‐well plate and processed with the required treatments. They were fixed in 4% formaldehyde (Biosharp) for 15 min and then blocked with 5% bovine serum albumin (BSA) (143,066, Biofroxx) for 1 h. They were incubated with ki67 antibody (Abcam) overnight at 4°C and then incubated with Cy3‐conjugated secondary antibody (Life Technologies) for 3 h at room temperature. Cell nuclei were labelled with 4′,6‐diamidino‐2‐phenylindole (DAPI, C1002, Beyotime). The images of stained cells were observed under a fluorescence microscope.

### 2.7 Cell migration assay

Cell migration ability was determined using a Transwell chamber (8.0 μm pores, Corning). Briefly, HRVECs were resuspended in a serum‐free DMEM medium. 100 μl cell suspension (1╳10^5^ cells) were added to the upper chamber and 400 μl normal complete medium was added to the lower chamber. The migrated cells were fixed in methanol and stained with 0.5% crystal violet solution (C805211, Macklin). These non‐migrated cells in the upper chamber were removed. The cells that passed the filter membrane were counted under a bright‐field microscope.

### 2.8 Tube formation assay

A precooled 24‐well plate was coated with the Growth Factor Reduced Matrigel (356234, BD Biosciences). After the required treatment, HRVECs were seeded on Matrigel at a cell density of 1 × 10^5^/well cells and incubated at 37°C in 5% CO_2_ and 95% humidity. After 8 h culture, tube formation ability was observed under a light microscope and quantified by Image J software.

### 2.9 RNA pull down assay

The 3′‐end biotinylated tRF‐1020 or control miRNA (RiboBio, 50 nM) were transfected into HRVECs for 12 h. The biotin‐coupled RNA complex was obtained by incubating cell lysates with the streptavidin‐coated magnetic beads (Life Technologies). The amount of DVL mRNA or GAPDH mRNA in the bound fraction was determined by qRT‐PCR assay.

### 2.10 Evans blue assay

Retinal vascular permeability was determined by Evans blue assay.^12^ Briefly, the mice were given intraperitoneal anaesthesia with ketamine (80 mg/kg) and xylazine (10 mg/kg). Evans blue dye (100 mg/mL) was injected into the femoral vein at the dosage of 45 mg/kg and 0.2 ml blood samples were collected. After the dye circulated for 1 h, the chest cavity was opened and perfused with citrate buffer (0.05 M, pH 3.5). After the perfusion, the eyes were enucleated and fixed in 4% paraformaldehyde for 30 min at room temperature. The retina was carefully dissected after removing the cornea, lens, and vitreous humour. For the quantitative assessment, the retina was incubated with formamide (Sigma‐Aldrich) overnight at 78°C and centrifuged at 4°C for 30 min. Evans blue dye in the supernatant was detected at the absorbance of 620 nm and 740 nm and compared with the blood samples treated similarly except for solubilization. The concentration of dye was calculated based on the standard curve of Evans blue in formamide and normalized to dry retina weight.

### 2.11 Retinal trypsin digestion assay

The eyes were enucleated and fixed in 4% paraformaldehyde for 24 h. The retinas were removed, gently shaken in water at room temperature overnight, and then incubated with 3% trypsin (BD, Difco) at 37 °C for 1 h. After trypsin digestion, the tissue was shaken gently to free vessel network, washed, and mounted on the glass slides. Dried retinal vessels were stained with periodic acid‐Schiff haematoxylin (PAS‐haematoxylin). Ten fields were randomly selected in each retina, and the number of acellular capillaries was counted in each field.^13^

### 2.12 Enzyme‐linked immunosorbent assay (ELISA)

ELISA assays were conducted to detect the levels of VEGF, IL‐6, and TNF‐α in retinal lysates. The levels of VEGF, IL‐6, and TNF‐α in the supernatant were detected by the enzyme‐linked immunosorbent assay (ELISA) kit (R&D Systems) according to the manufacturer's instructions. Finally, optical density was determined at a 450 nm wavelength and plotted according to the numerical values.

### 2.13 Clinical samples collection

Aqueous humour samples were harvested from the patients with diabetes retinopathy (DR, *n* = 20 eyes) and cataract (non‐DR, *n* = 20 eyes). Vitreous samples were harvested from the subjects with idiopathic macular hole (non‐DR, *n* = 20 eyes) and PDR (*n* = 20 eyes) during pars plana vitrectomy. These clinical samples were collected in tubes, placed immediately on ice, centrifuged for 20 min, and stored at −80 °C until use. The patients with cancers, serious infection, and active chronic inflammatory diseases were excluded.

### 2.14 Statistical analysis

Data were first determined for the normality using the D'Agostino Pearson omnibus normality test. For normally‐distributed data with equal variance, significant difference was evaluated by Student *t*‐test (2‐group comparison) or 1‐way anova followed by the post hoc Bonferroni test (multi‐group comparison). For non‐normally‐distributed data or data with unequal variances, a significant difference was evaluated by a nonparametric Mann–Whitney *U* test (2‐group comparison) or Kruskal–Wallis test followed by the post hoc Bonferroni test (multi‐group comparison). *p* < 0.05 was considered significant. All statistical analysis was conducted using GraphPad prism version 6.0.

## 3 RESULTS

### 3.1 The expression level of tRF‐1020 is significantly down‐regulated upon diabetic stress in vitro and in vivo

**FIGURE 1:**
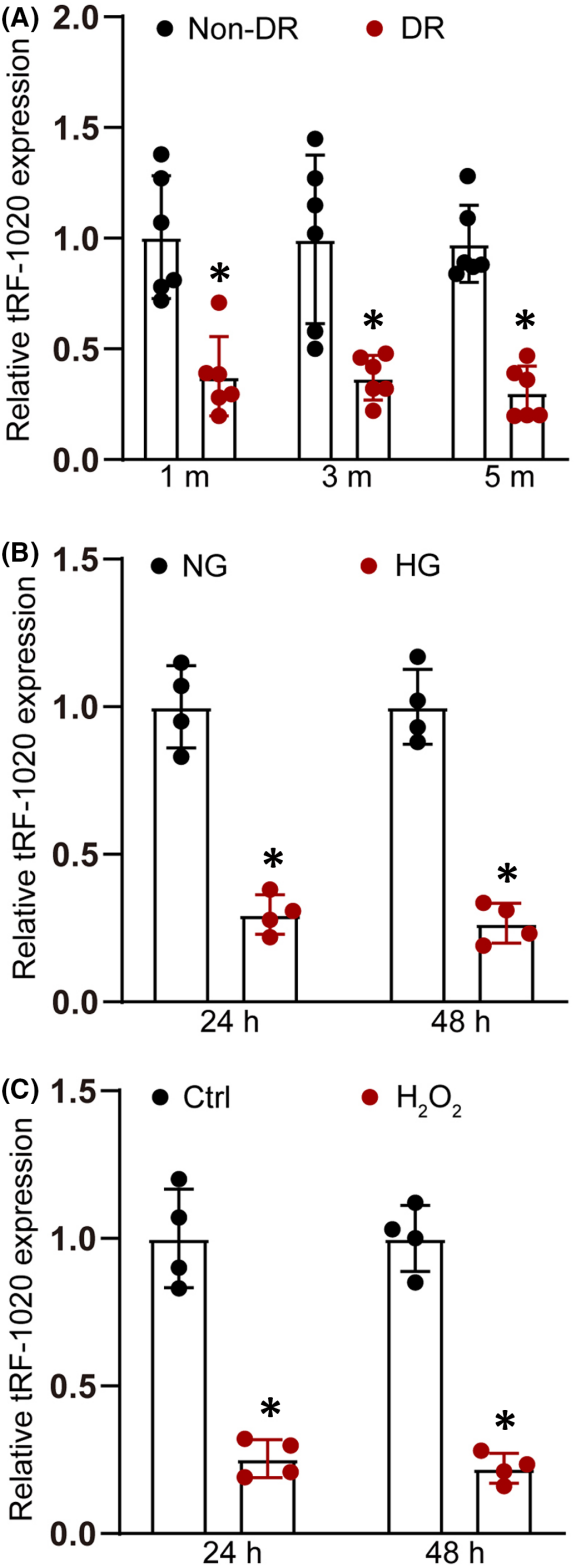
The expression level of tRF‐1020 is significantly down‐regulated upon diabetic stress in vitro and in vivo. (A) qRT‐PCRs were conducted to detect tRF‐1020 expression in retinal vessels isolated from nondiabetic retinas and diabetic retinas at 1 month, 3 months, and 5 months after the induction of diabetes (*n* = 6 animals per group, *p < 0.05 vs. Non‐DR group, Student's *t*‐test). (B) qRT‐PCRs were conducted to detect tRF‐1020 expression in HRVECs cultured in the medium containing normal glucose (NG, 5.55 mM) and high glucose (HG, 25 mM) for 24 h and 48 h (*n* = 4, *p < 0.05 vs. NG group, Student's *t*‐test). (C) qRT‐PCRs were conducted to detect the levels of tRF‐1020 expression in HRVECs cultured in the medium without (Ctrl) or with H_2_O_2_ (50 μm) for 24 h and 48 h (*n* = 4, *p < 0.05 vs. H_2_O_2_ group, Student's *t*‐test).

Diabetes was induced by intraperitoneal injection of streptozotocin (STZ) for five consecutive days using the 8‐week‐old C57BL/6J mice. Retinal vessels were extracted from the mouse retinas at 1 month, 3 months, and 5 months after diabetes induction. qRT‐PCR assays revealed that the levels of tRF‐1020 expression were significantly down‐regulated in diabetic retinal vessels compared with that in the nondiabetic controls (Figure 1A). Retinal vascular endothelial cells (HRVECs) were exposed to high glucose or oxidative stress to mimic diabetic stress in vitro. Compared with the control group, high glucose (25 mM) or oxidative stress (H_2_O_2_, 50 μm) led to decreased levels of tRF‐1020 expression in HRVECs after 24‐hour or 48‐hour treatment (Figure 1B,C).

### 3.2 tRF‐1020 regulates endothelial angiogenic function in vitro

**FIGURE 2:**
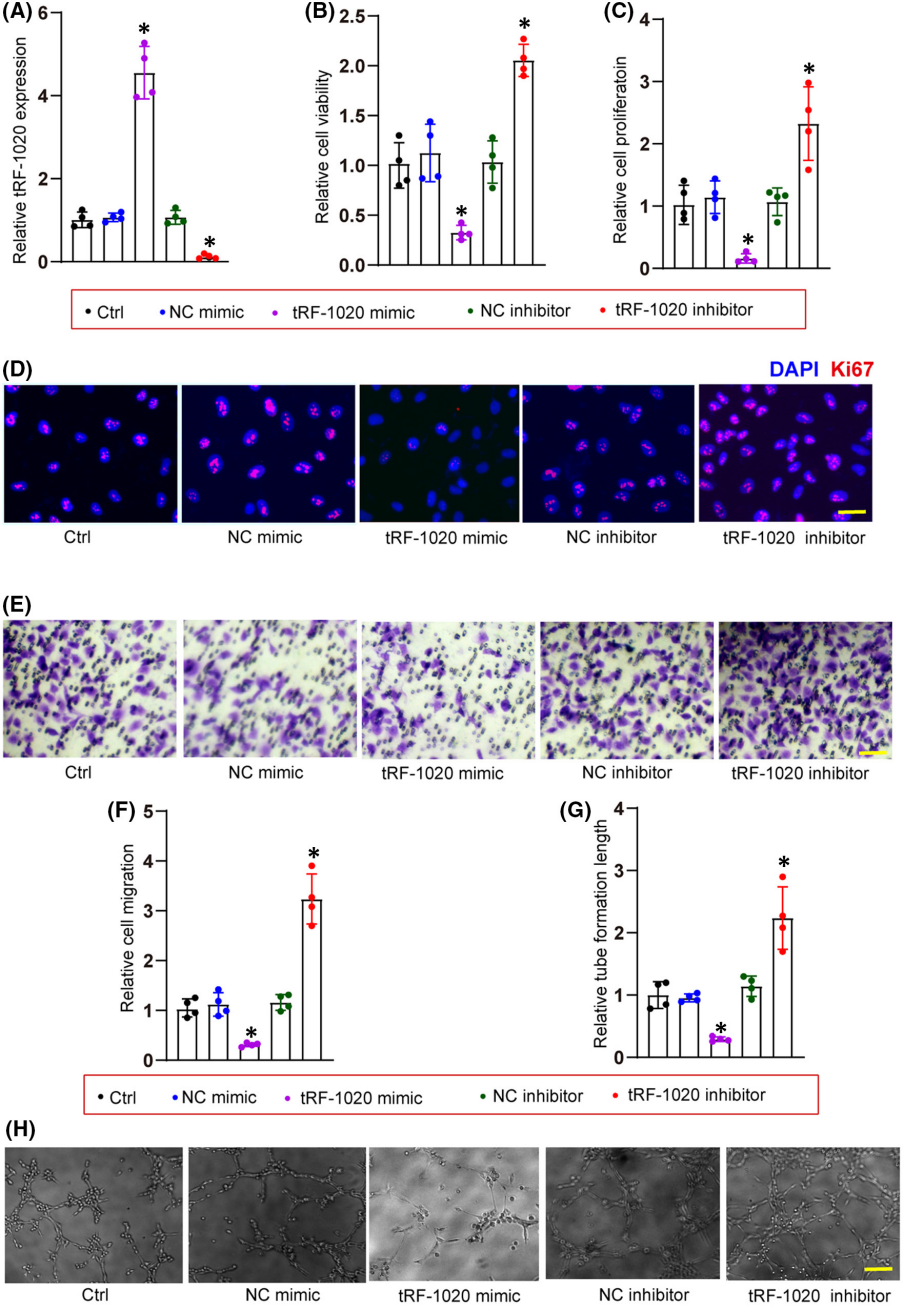
tRF‐1020 regulates endothelial angiogenic function in vitro. (A) HRVECs were transfected with negative control (NC) mimic, tRF‐1020 mimic, tRF‐1020 inhibitor, negative control (NC) inhibitor, or left untreated (Ctrl) for 48 h. qRT‐PCRs were conducted to detect the expression levels of tRF‐1020 (*n* = 4, *p < 0.05 vs. Ctrl group). (B) Cell viability was determined by CCK‐8 assay (*n* = 4, *p < 0.05 vs. Ctrl group). (C and D) Cell proliferation was determined by Ki67 staining (*n* = 4, *p < 0.05 vs. Ctrl group). A representative image along with the quantification result was shown. Scale bar: 20 μm. (E and F) Transwell assay was conducted to determine HRVEC migration (*n* = 4, *p < 0.05 vs. Ctrl group). A representative image along with the quantification result was shown. Scale bar: 50 μm. (G and H) The tube‐like structures were observed at 8 h after seeding HRVECs on the Matrigel matrix. Average tube length for each field was statistically analysed (*n* = 4, *p < 0.05 vs. Ctrl group). Scale bar: 100 μm. The significant difference was evaluated by 1‐way anova followed by the Bonferroni post hoc test.

We then determined whether tRF‐1020 regulated endothelial biology in vitro. tRF‐1020 mimics or inhibitors were transfected into HRVECs to determine the potential function of tRF‐1020 in endothelial cells. The transfection of tRF‐1020 mimics led to enhanced levels of tRF‐1020. By contrast, the transfection of tRF‐1020 inhibitors led to reduced levels of tRF‐1020 (Figure 2A).

CCK‐8 assays showed that the transfection of tRF‐1020 mimics significantly decreased the viability of HRVECs (Figure 2B). Ki67 staining assays showed that the transfection of tRF‐1020 mimics reduced the proliferation ability of HRVECs (Figure 2C,D). Transwell migration assays showed that the transfection of tRF‐1020 mimics decreased the migratory ability of HRVECs (Figure 2E,F). Tube formation assays showed that the relative tube length was significantly decreased in tRF‐1020 mimic‐transfected group compared with the control group (Figure 2G,H). By contrast, the transfection of tRF‐1020 inhibitors led to increased cell viability, proliferative ability, migration ability, and tube formation ability (Figure 2B–H). Taken together, the above‐mentioned evidence suggests that tRF‐1020 is an important regulator of endothelial angiogenic functions.

### 3.3 tRF‐1020 regulates diabetes‐induced retinal vascular dysfunction in vivo

**FIGURE 3:**
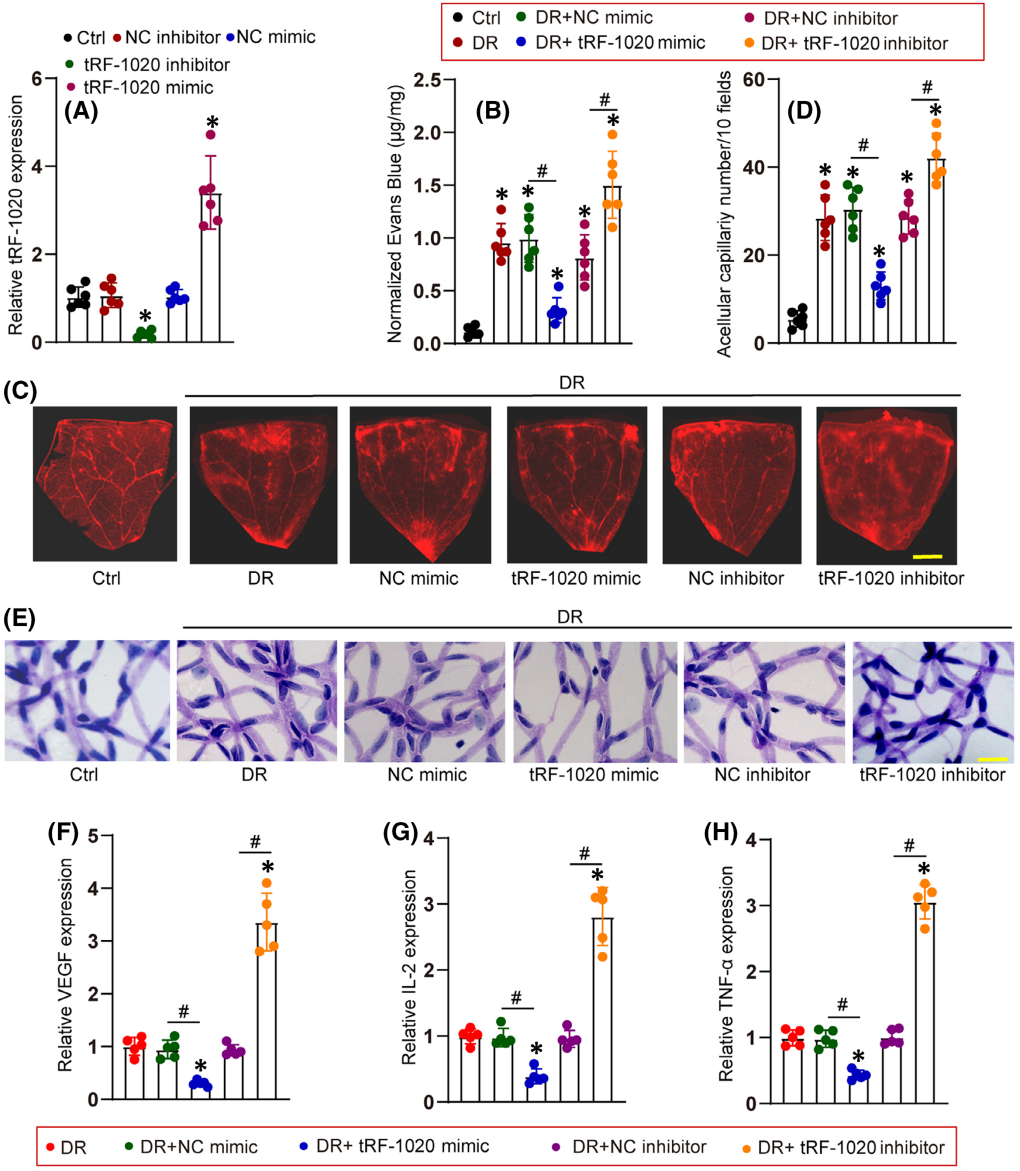
tRF‐1020 regulates diabetes‐induced retinal vascular dysfunction in vivo. (A) C57BL/6 mice received an intravitreous injection of negative control (NC) inhibitors, tRF‐1020 inhibitors, negative mimics, tRF‐1020 mimics, or left untreated (Ctrl) for 2 weeks. qRT‐PCR assays were conducted to determine the levels of tRF‐1020 expression (*n* = 6, *p < 0.05 vs. Ctrl group). (B and C) The mice were infused with Evans blue dye for 3 hours. The fluorescence signal of flat‐mounted retinas was observed. A representative image along with the quantification of Evans blue leakage was shown. Scale bar: 500 μm. The red fluorescent is Evans blue signalling. (D and E) Retinal trypsin digestion was used to detect the number of acellular retinal capillaries. Acellular capillaries were counted in 10 random fields per retina and averaged. Scale bar, 50 μm. For B‐E: *n* = 6, *p < 0.05 vs. non‐DR Ctrl group; ^#^ *p* < 0.05 between the marked groups. (F‐H) ELISA assays were conducted to determine the amount of VEGF, IL‐2, and TNF‐α in retinal lysates (*n* = 5; *p < 0.05 vs. DR group; ^#^ *p* < 0.05 between the marked groups). The significant difference was evaluated by the Kruskal‐Wallis test followed by the post hoc Bonferroni test.

We further determined the role of tRF‐1020 in diabetes‐induced retinal vascular dysfunction in vivo. Diabetic C57BL/6 mice received an intravitreous injection of tRF‐1020 mimics or inhibitors to regulate the levels of tRF‐1020. Injection of tRF‐1020 mimics led to increased levels of tRF‐1020, while injection of tRF‐1020 inhibitors led to reduced levels of tRF‐1020 (Figure 3A).

Evans blue assays demonstrated that compared with diabetic retinas, the injection of tRF‐1020 mimics alleviated diabetes‐induced retinal vascular leakage (Figure 3B,C). The acellular vessel is an important pathological feature of diabetic retinas. Trypsin digestion assays revealed that injection of tRF‐1020 mimics decreased the number of acellular vessels in diabetic retinas (Figure 3D,E). ELISA assays showed that the injection of tRF‐1020 mimics alleviated diabetes‐induced retinal inflammation as shown by decreased expression of VEGF, interleukin (IL)‐2, and TNF‐α (Figure 3F–H). By contrast, injection of tRF‐1020 inhibitors aggravated diabetes‐induced retinal vascular leakage, increased the number of acellular vessels in diabetic retinas, and aggravated diabetes‐induced retinal inflammation (Figure 3B–H).

### 3.4 tRF‐1020 directly regulates DVL‐2 expression in endothelial cells

**FIGURE 4:**
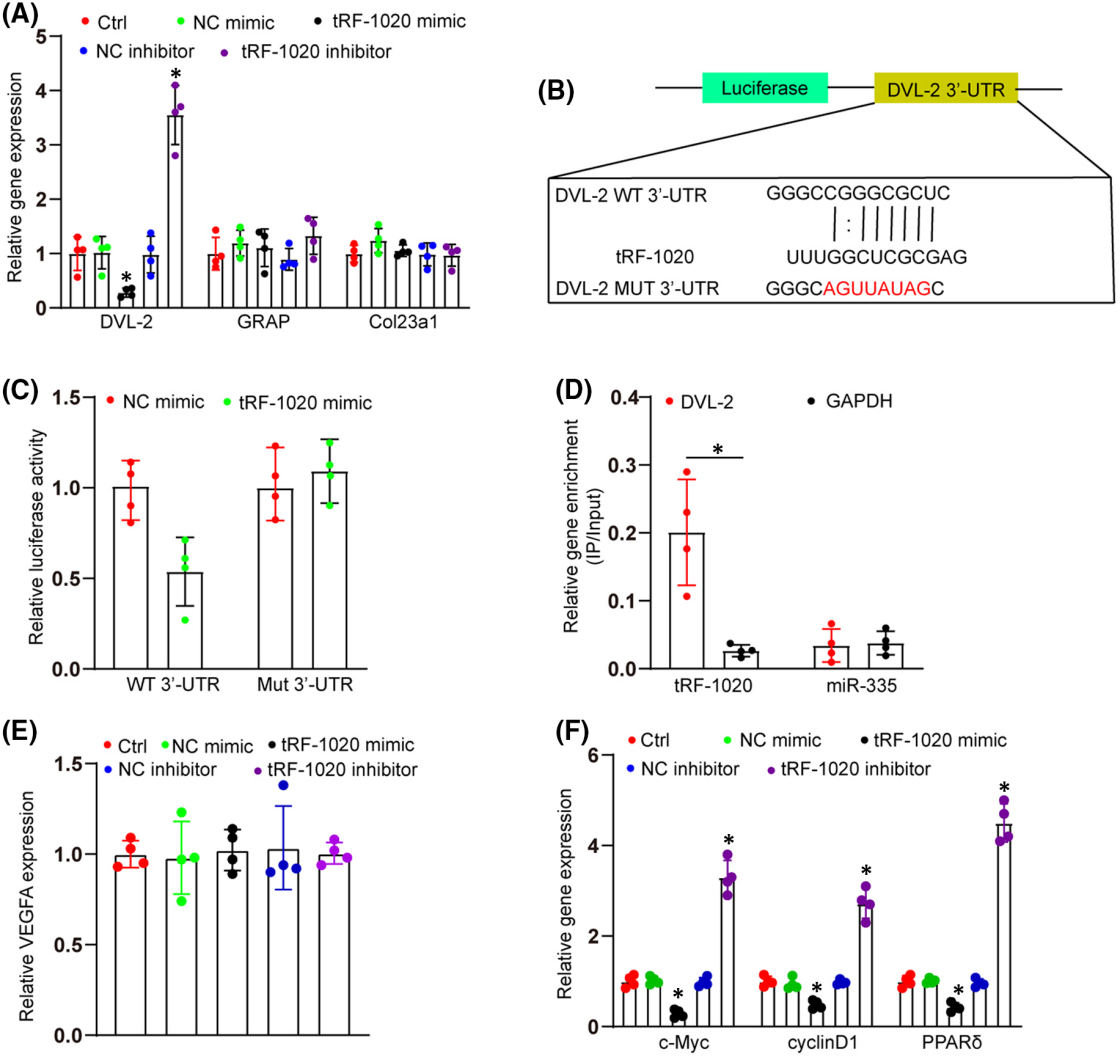
tRF‐1020 directly regulates DVL‐2 expression in endothelial cells. (A) The expression levels of DVL‐2, GRAP, and Col23a1 were determined by qRT‐PCRs in HRVECs after the transfection of tRF‐1020 inhibitor, negative control (NC) inhibitor, tRF‐1020 mimic, negative control (NC) mimic, or left untreated (Ctrl) (*n* = 4, *p < 0.05 vs. Ctrl group). (B) 3′‐UTR fragment of wide‐type (WT) and mutated DVL‐2 which disrupted the interaction with tRF‐1020. MUT: mutated DVL‐2 3’‐UTR without tRF‐1020‐binding sites. (C) The luciferase activity of wild‐type DVL‐2 3’‐UTR or mutant DVL‐2 3’‐UTR after transfection with tRF‐1020 mimic or NC mimic in HRVECs were determined (*n* = 4, *p < 0.05 vs. tRF‐1020 mimic group). (D) The 3′‐end biotinylated tRF‐1020 or biotinylated miR‐335 were transfected into HRVECs. After streptavidin capture, the levels of DVL‐2 and GAPDH in the input and bound fractions were detected by qRT‐PCR assays. The relative immunoprecipitate (IP)/input ratios were plotted (*n* = 4, *p < 0.05 vs. DVL‐2 group). (E) The expression levels of VEGFA were determined by qRT‐PCRs in HRVECs after transfection of tRF‐1020 inhibitor, negative control (NC) inhibitor, tRF‐1020 mimic, negative control (NC) mimic, or left untreated (Ctrl) (*n* = 4, *p < 0.05 vs. Ctrl group). (F) The expression levels of c‐Myc, cyclin D1, and PPARδ were determined by qRT‐PCRs in HRVECs after transfection of tRF‐1020 inhibitor, negative control (NC) inhibitor, tRF‐1020 mimic, negative control (NC) mimic, or left untreated (Ctrl) (*n* = 4, *p < 0.05 vs. Ctrl group). The significant difference was evaluated by 1‐way anova followed by the Bonferroni post hoc test.

We next explored the molecular mechanism of tRF‐1020 in endothelial cells. The potential target genes of tRF‐1020 were predicted by TargetScan and miRanda databases according to the presence of binding sites in the 3′‐UTR. To reduce the prediction scope, we evaluated the overlapping gene results from TargetScan and miRanda databases. We selected DVL‐2, GRAP, and Col23a1 for the subsequent study due to their roles in regulating angiogenic effects. qRT‐PCR assays showed that the expression level of DVL‐2 was significantly decreased after the transfection of tRF‐1020 mimics. By contrast, the transfection of tRF‐1020 inhibitors led to an increased expression level of DVL‐2. However, the transfection of tRF‐1020 inhibitors or mimics did not alter the expression levels of GRAP and Col23a1 (Figure 4A). We further conducted the luciferase assays to verify the direct binding between tRF‐1020 and DVL‐2. The binding sites for tRF‐1020 on the wild‐type DVL‐2 3′‐UTR are shown in Figure 4B. The luciferase activity of wild‐type DVL‐2 3′‐UTR was significantly reduced after transfection of tRF‐1020 mimic but markedly increased after transfection of tRF‐1020 inhibitor. However, altered tRF‐1020 expression did not affect the luciferase activity of mutant DVL‐2 3’‐UTR (Figure 4C). RNA pull down assays were conducted to detect the interaction between tRF‐1020 and DVL‐2. We observed a greater enrichment of DVL‐2 in tRF‐1020‐captured fraction in comparison with the negative control, biotinylated miR‐335 (Figure 4D). VEGFA is shown as a key regulator during retinal vascular dysfunction. The results show that tRF‐1020 intervention did not alter the expression levels of VEGFA, suggesting that tRF‐1020 did not have off‐sites or non‐specific targets (Figure 4E). DVL‐2 is an important regulator of Wnt/β‐catenin signalling. We next investigated whether tRF‐1020 intervention affected the expression of the downstream genes of Wnt signalling, such as c‐Myc, cyclin D1, and peroxisome proliferator‐activated receptor (PPAR) δ. The results showed that tRF‐1020 mimics reduced the expression levels of c‐Myc, cyclinD1, and PPARδ, whereas tRF‐1020 inhibitors decreased the expression levels of c‐Myc, cyclinD1, and PPARδ (Figure 4F). Collectively, these results indicate that tRF‐1020 directly regulates DVL‐2 expression in endothelial cells.

### 3.5 tRF‐1020 regulates endothelial angiogenic function by targeting DVL‐2

**FIGURE 5:**
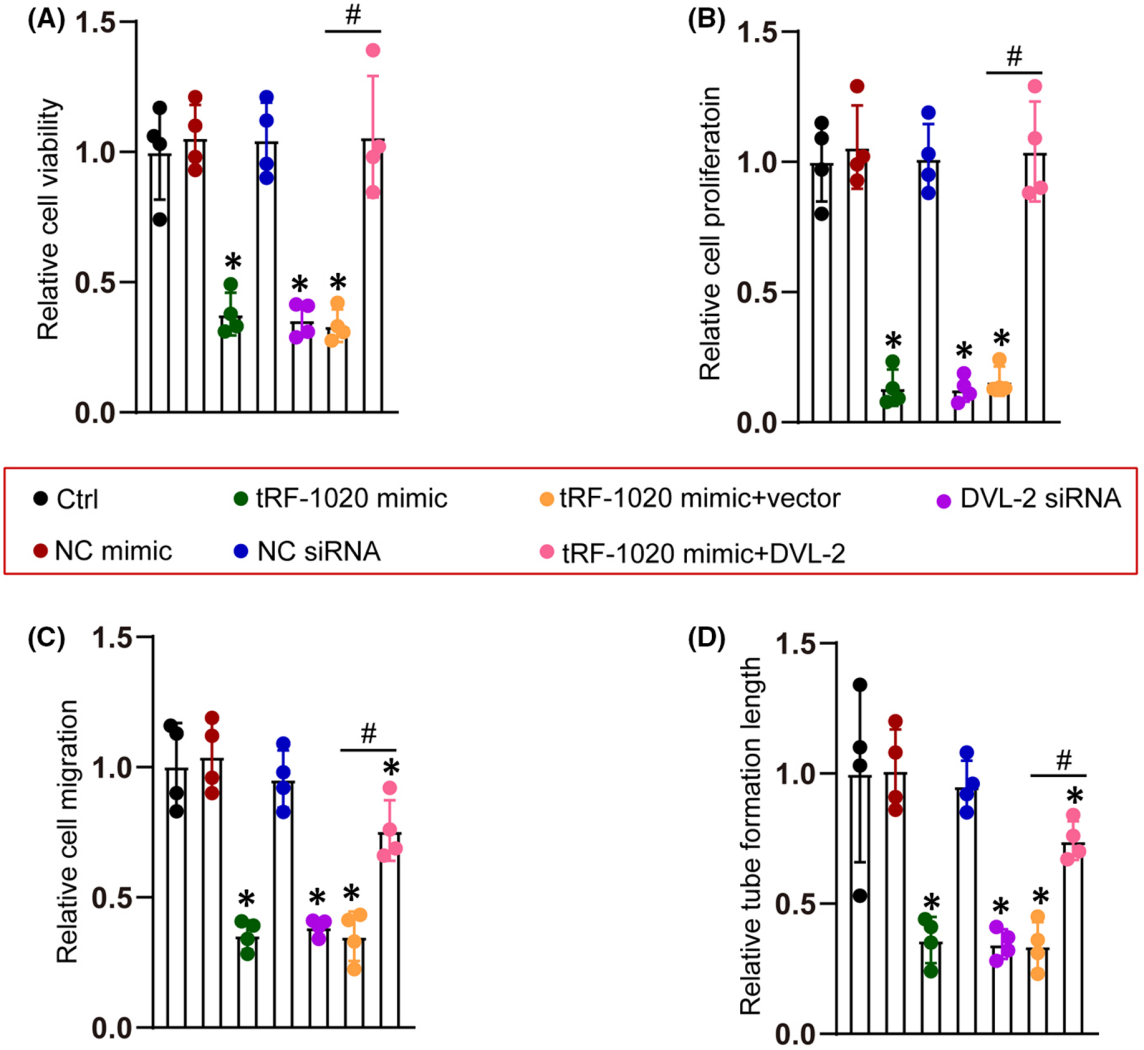
tRF‐1020 regulates endothelial angiogenic function by targeting DVL‐2. (A–D) HRVECs were treated as shown for 48 h. Cell viability was determined by CCK‐8 assay (A, *n* = 4). Cell proliferation was determined by Ki67 staining and the quantification result was shown (B, *n* = 4). Transwell assay was conducted to determine HRVEC migration and the quantification result was shown (C, *n* = 4). The tube‐like structures were observed at 8 h after seeding HRVECs onto the Matrigel matrix. The average tube length for each field was statistically analysed (D, *n* = 4). *p < 0.05 vs. Ctrl group, ^#^ *p* < 0.05 tRF‐1020 + vector vs. tRF‐1020 + DVL‐2. The significant difference was evaluated by 1‐way anova followed by the Bonferroni post hoc test.

We further conducted the rescue experiments to determine whether tRF‐1020 regulated endothelial function by targeting DVL‐2 in HRVECs. Transfection of tRF‐1020 mimic led to decreased cell viability, proliferation ability, migration ability, and tube formation ability of HRVECs. DVL‐2 knockdown could achieve the similar effects as tRF‐1020 mimic on endothelial angiogenic effects (Figure 5A–D). However, DVL‐2 overexpression could partially reversed the inhibitory effects of tRF‐1020 mimic on endothelial angiogenic effects, which could lead to increased cell viability, proliferation ability, migration ability, and tube formation ability compared with tRF‐1020 mimic group (Figure 5A–D).

### 3.6 tRF‐1020 is a potential biomarker for diabetic retinopathy

**FIGURE 6:**
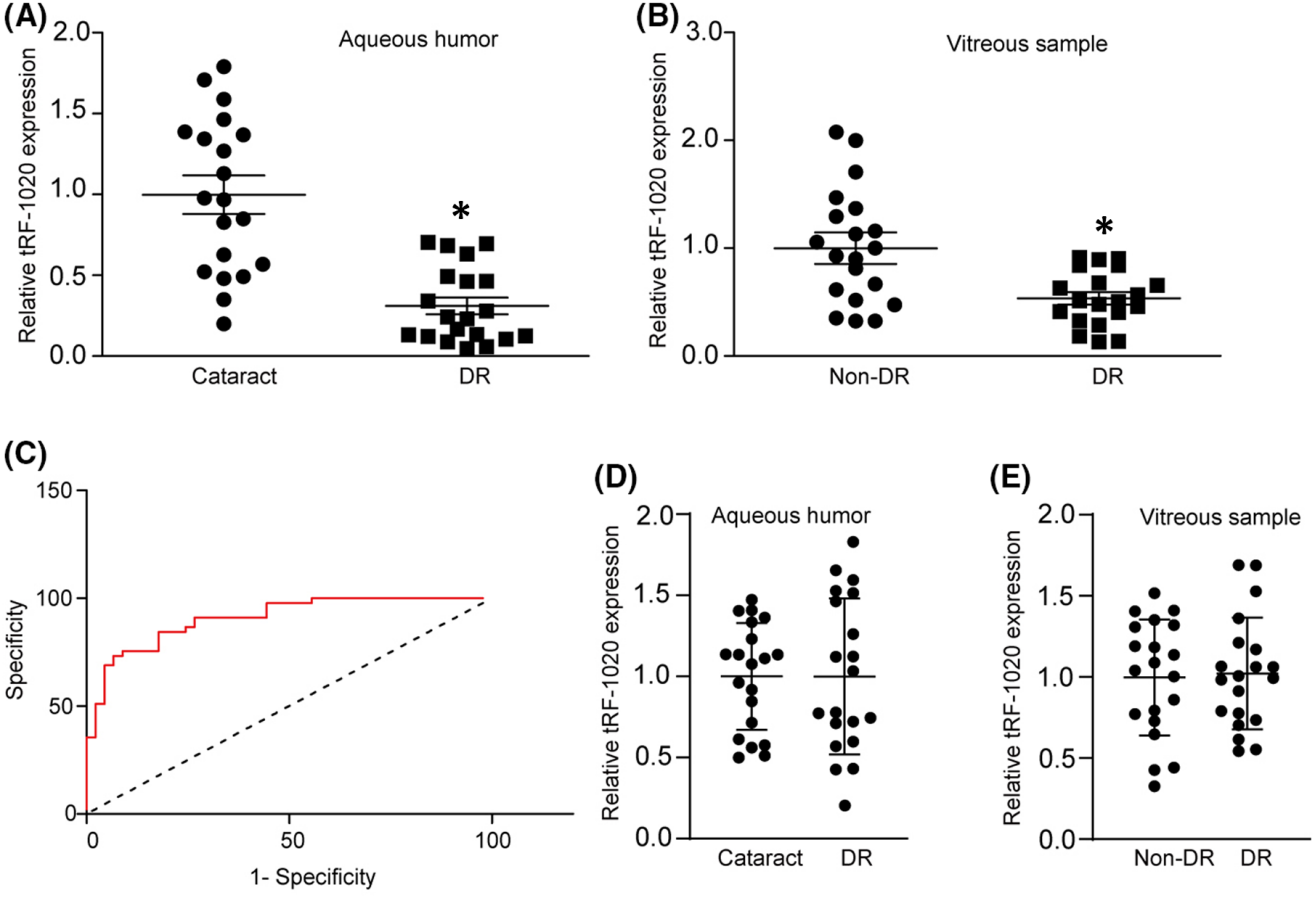
tRF‐1020 is a potential biomarker for diabetic retinopathy. (A) qRT‐PCR assays were conducted to detect the levels of tRF‐1020 in aqueous humour samples of the patients with diabetic retinopathy (DR, *n* = 20 eyes) and cataract (*n* = 20 eyes). (B) qRT‐PCR assays were conducted to detect the levels of tRF‐1020 in vitreous samples of the patients with diabetic retinopathy (DR, *n* = 20 eyes) and macular holes (non‐DR, *n* = 20 eyes). (C) Receiver operating characteristic (ROC) curve analysis of tRF‐1020 and their corresponding area under the curve (AUC) values in the diagnostic performance for DR. (D) qRT‐PCR assays were conducted to detect the levels of tRF‐1040 in aqueous humour samples of the patients with diabetic retinopathy (DR, *n* = 20 eyes) and cataract (*n* = 20 eyes). (E) qRT‐PCR assays were conducted to detect the levels of tRF‐1040 in vitreous samples of the patients with diabetic retinopathy (DR, *n* = 20 eyes) and macular holes (non‐DR, *n* = 20 eyes). *p < 0.05 vs. non‐DR group. The significant difference was evaluated by 1‐way anova followed by the Bonferroni post hoc test.

We next evaluated the diagnostic value of tRF‐1020 for DR. Aqueous humour is an important body fluid in the eye, which is known to be related to various ocular diseases.^14^ The baseline demographics of the patients for aqueous humour collection is shown in Table 1. The levels of tRF‐1020 were significantly down‐regulated in the aqueous humour samples of the patients with DR, but not in other patients with cataract (Figure 6A). We then determined the expression levels of tRF‐1020 in the vitreous samples of the patients with DR and non‐diabetic controls. The baseline demographics of the patients for vitreous sample collection is shown in Table 2. qRT‐PCR assays showed that the levels of tRF‐1020 were significantly down‐regulated in the vitreous samples of the patients with DR (Figure 6B). To evaluate the discriminative power of tRF‐1020 between non‐DR controls and the patients with DR, the area under the ROC‐AUC was calculated. The ROC‐AUC for tRF‐1020 for differentiating the patients with DR from non‐DR controls was 0.9086 (Figure 6C; 95% CI: 0.8537–0.9712; sensitivity 0.8963, specificity 0.8053), suggesting that tRF‐1020 is a promising marker for the diagnosis of DR. We also determined the expression levels of tRF‐1040 in the aqueous humour and vitreous samples of the patients with DR and non‐diabetic controls. The results showed that the levels of tRF‐1040 expression did not show significant difference between the patients with DR and non‐DR patients in the aqueous humour and vitreous samples (Figure 6D,E), suggesting that tRF‐1020 is a specific biomarker for the diagnosis of DR.

**TABLE 1:** Baseline demographics of the patients for aqueous humour collection

Characteristics	DR (*n* = 20)	Cataract (*n* = 20)
Age (years), mean ± SD	61.2 ± 8.5	63.1 ± 9.3
Gender (male/female)	14/6	12/8
Duration of diabetes (years)	10–16 years	N.A.
IOP (mmHg)	15 ± 3.9	14.8 ± 4.3
Blood glucose level (mM)	9.2 ± 2.9	4.8 ± 1.2

**TABLE 2:** Baseline demographics of the patients for vitreous sample collection

Characteristics	DR (*n* = 20)	Macular hole (*n* = 20)
Age (years), mean ± SD	61.6 ± 6.2	60.2 ± 5.7
Gender (male/female)	15/5	10/10
Duration of diabetes (years)	12–20 years	N.A.
IOP (mmHg)	14.3 ± 3.2	15.7 ± 2.1
Blood glucose level (mM)	8.9 ± 2.1	4.1 ± 1.5

## 4 DISCUSSION

DR remains a leading cause of vision loss in the working‐age population. Its pathogenesis is highly complicated, in which vascular, inflammatory, and neuronal mechanisms are involved. Vascular complication mediates structural and molecular changes in DR.^4^, ^15^ However, the molecular mechanisms underlying retinal microvascular complications are not completely characterized. In this study, we demonstrate that tRF‐1020 is significantly down‐regulated in diabetic retinal vessels and retinal endothelial cells following high glucose or H_2_O_2_ stress. tRF‐1020 mimic suppresses endothelial angiogenic functions and alleviates diabetes‐induced retinal vascular complications by targeting DVL‐2‐mediated Wnt signalling. The levels of tRF‐1020 are significantly down‐regulated in the aqueous humour and vitreous samples of DR patients. Collectively, tRF‐1020 plays an important role in DR and is a potential target for the prevention/treatment of DR.

Diabetes‐induced retinal vascular complications are the major causes of blindness. Endothelial cells are the constitutive part of the vasculature and are involved in the regulation of angiogenesis, haemostasis, and vascular tone.^16^ Hyperglycaemia leads to decreased levels of tRF‐1020 in endothelial cells and retinal vessels. Reduced levels of tRF‐1020 can induce abnormal proliferation, migration, and activation of endothelial cells. In the diseased condition, the unceasing or excessive proliferation and migration of endothelial cells occur in retinal microvascular complications.^15^ Reduced levels of tRF‐1020 aggravate diabetes‐induced retinal vascular complications as shown by increased vascular leakage, acellular capillary number, and inflammation response. We thus speculate that reduced tRF‐1020 level is a predisposing factor of diabetes‐induced retinal vascular complication.

tRNA‐derived small RNAs (tsRNAs), including tRNA‐derived fragments (tRFs) and tRNA‐derived stress‐induced RNAs (tiRNAs), can provide additional regulatory layers of gene expression. Accumulating evidence has shown that tsRNAs play important roles in RNA silencing through the complementation between tsRNAs and target mRNAs.^17^ In addition, some tsRNAs are associated with Argonaute proteins and regulate gene expression in an RNA interference (RNAi) manner.^18^ Previous studies have shown that tRF‐3019a directly regulates FBXO47 expression via binding a site in the 3′‐UTR and Ago2.^19^ 5′‐tiRNA^Val^ inhibits the FZD3‐mediated Wnt/β‐catenin signalling pathway in breast cancer cells via binding to the 3′‐UTR of FZD3.^20^ Herein, RNA pull down assay, luciferase activity assay, and qRT‐PCR assay indicate that tRF‐1020 exerts its biological role via a similar mechanism. It can directly bind to the 3′‐UTR of DVL‐2 and lead to the inhibition of DVL‐2 expression.

Molecular mechanism study has revealed that tRF‐1020 directly regulates DVL‐2 expression in endothelial cells. DVL‐2 belongs to the dishevelled family and is a key regulator of Wnt/β‐catenin signalling.^21^ A high level of DVL‐2 can also efficiently activate Wnt/β‐catenin signalling. Increased DVL‐2 expression can aid in β‐catenin release from cytosolic Axin/GSK‐3/APC complex.^22^, ^23^ Moreover, DVL‐2 can mediate the formation of the DVL/c‐Jun/β‐catenin/TCF functional complex, leading to the stabilization of β‐catenin‐TCF interaction.^24^ All events can ultimately trigger the activation of Wnt/β‐catenin target genes. Dysregulation of Wnt signalling can mediate pathological vascular growth in proliferative retinopathy.^25^ In this study, the transfection of tRF‐1020 mimic leads to decreased levels of DVL‐2. By contrast, transfection of tRF‐1020 inhibitor leads to increased levels of DVL‐2. Altered expression of tRF‐1020 could affect the activation of Wnt signalling. This regulatory mechanism provides a novel insight into retinal microvascular complications.

## 5 CONCLUSION

In conclusion, this study demonstrates that tRF‐1020 is an important player in the progression of DR. tRF‐1020 shows great potentials for regulating endothelial angiogenic functions and treating diabetes‐induced retinal microvascular complications. Clinical data show that the levels of tRF‐1020 expression are down‐regulated in the aqueous humour and vitreous samples of DR, which can differentiate DR patients from non‐DR controls. As a result, tRF‐1020 is an appealing therapeutic target for the diagnosis and treatment of DR.

## AUTHOR CONTRIBUTIONS

**jianling du:** Conceptualization (equal); data curation (equal); formal analysis (equal); funding acquisition (equal); project administration (equal); resources (equal). **cong ma:** Data curation (equal); formal analysis (equal); software (equal); supervision (equal); validation (equal); visualization (equal). **xiang Ma:** fuding aquisition (equal); project administration (equal).

## CONFLICT OF INTEREST

The authors declare that there are no competing interests associated with the manuscript.

## Data Availability

The data that support the findings of this study are available from the corresponding author upon reasonable request.
